# Substrates of the chloroplast small heat shock proteins 22E/F point to thermolability as a regulative switch for heat acclimation in *Chlamydomonas reinhardtii*

**DOI:** 10.1007/s11103-017-0672-y

**Published:** 2017-11-01

**Authors:** Mark Rütgers, Ligia Segatto Muranaka, Timo Mühlhaus, Frederik Sommer, Sylvia Thoms, Juliane Schurig, Felix Willmund, Miriam Schulz-Raffelt, Michael Schroda

**Affiliations:** Molekulare Biotechnologie & Systembiologie, TU Kaiserslautern, Paul-Ehrlich Straße 23, 67663 Kaiserslautern, Germany

**Keywords:** Molecular chaperones, Protein homeostasis, Chloroplast, Protein–protein interactions, Mass spectrometry

## Abstract

**Key message:**

We have identified 39 proteins that interact directly or indirectly with high confidence with chloroplast HSP22E/F under heat stress thus revealing chloroplast processes affected by heat.

**Abstract:**

Under conditions promoting protein unfolding, small heat shock proteins (sHsps) prevent the irreversible aggregation of unfolding proteins by integrating into forming aggregates. Aggregates containing sHsps facilitate the access of Hsp70 and ClpB/Hsp104 chaperones, which in ATP-dependent reactions disentangle individual proteins from the aggregates and assist in their refolding to the native state. *Chlamydomonas reinhardtii* encodes eight different sHsps (HSP22A to H). The goal of this work was to identify chloroplast-targeted sHsps in Chlamydomonas and to obtain a comprehensive list of the substrates with which they interact during heat stress in order to understand which chloroplast processes are disturbed under heat stress. We show that HSP22E and HSP22F are major chloroplast-targeted sHsps that have emerged from a recent gene duplication event resulting from the ongoing diversification of sHsps in the *Volvocales*. HSP22E/F strongly accumulate during heat stress and form high molecular mass complexes. Using differential immunoprecipitation, mass spectrometry and a stringent filtering algorithm we identified 39 proteins that with high-confidence interact directly or indirectly with HSP22E/F under heat stress. We propose that the apparent thermolability of several of these proteins might be a desired trait as part of a mechanism enabling Chlamydomonas chloroplasts to rapidly react to thermal stress.

**Electronic supplementary material:**

The online version of this article (doi:10.1007/s11103-017-0672-y) contains supplementary material, which is available to authorized users.

## Introduction

Small heat shock proteins (sHsps) are ancient proteins characterized by a core α-crystallin domain of about 100 amino acids that is flanked by an N-terminal arm of variable length and sequence and a short C-terminal extension (Haslbeck and Vierling 2015). sHsps form dimers as basic building block that may assemble further into barrel-like structures of 12 to more than 32 subunits (Fleckenstein et al. 2015; Kim et al. 1998; van Montfort et al. 2001). Under conditions promoting protein unfolding, sHsps prevent the irreversible aggregation of unfolding proteins by integrating into forming aggregates. Aggregates containing sHsps facilitate the access of Hsp70 and ClpB/Hsp104 chaperones, which in ATP-dependent reactions disentangle individual proteins from the aggregates and assist in their refolding to the native state (Cashikar et al. 2005; Lee and Vierling 2000; Mogk et al. 2003a, b). In land plants, sHsps are targeted to the cytosol, the ER, peroxisomes, mitochondria and chloroplasts (Waters 2013). Chloroplast sHsps have been shown to protect photosystem II (PSII) against oxidative stress (Harndahl et al. 1999; Kim et al. 2012). In addition, the chloroplast sHsp Hsp21 has been suggested to play a role in the conversion of chloroplasts to chromoplasts during tomato fruit maturation (Neta-Sharir et al. 2005). Moreover, Hsp21 has been demonstrated to localize to nucleoids and to stabilize the plastid-encoded RNA polymerase complex under heat stress (Zhong et al. 2013). Hsp21 also has been shown to enhance survival rates of heat-stressed Arabidopsis plants and to maintain the integrity of thylakoid membrane protein complexes in heat-stressed, chlorophyll *b*-deficient *gun5* mutant plants (Chen et al. 2017).

Comprehensive lists of sHsp substrates have been obtained only for a few organisms/organelles, including *E. coli, Synechocystis*, yeast, *D. radiodurans, C. elegans*, and maize chloroplasts (Basha et al. 2004; Bepperling et al. 2012; Fleckenstein et al. 2015; Fu et al. 2013; Haslbeck et al. 2004; Hu et al. 2015). The goal of this work was to generate such a comprehensive list for a chloroplast-targeted sHsp in heat-stressed *Chlamydomonas reinhardtii* cells in order to understand which chloroplast processes are affected when heat stress affects chloroplast protein homeostasis. We focus here on the HSP22E/F proteins as they were predicted to be chloroplast-localized (Schroda and Vallon 2009) and, together with cytosolic HSP22A, were the only sHsps that ranked among the 280 most abundant proteins in heat-stressed Chlamydomonas cells (Schroda et al. 2015).

## Materials and methods

### Strains and culture conditions

*Chlamydomonas reinhardtii* strains cw15-302 (cw_d_, mt^+^, *arg7* ^−^, *nit* ^−^) and cw15-325 (cw_d_, mt^+^, *arg7* ^−^, *nit* ^+^) were kindly provided by R. Matagne (University of Liège, Belgium). Cells were grown on a rotatory shaker at 25 °C and ~ 50 μmol photons m^2^ s^− 1^ under mixotrophic conditions in Tris–acetate–phosphate (TAP) medium (Kropat et al. 2011). Cultures were diluted 1 day before and experiments were performed with mid–log phase cultures (about 4 × 10^6^ cells/ml). Cell densities were determined using a Z2 Coulter Counter (Beckman Coulter). For the heat stress experiments, cells were pelleted by centrifugation at 25 °C and 1300 g for 2 min, resuspended in pre-warmed TAP medium, and incubated in a water bath under agitation and constant illumination at ~ 50 µmol photons m^− 2^ s^− 1^.

### Cloning, expression and purification of recombinant HSP22F, HSP70A, CPN60B2, RbcL and TIG1

The HSP22F coding sequence was amplified from EST clone BP092687 (Asamizu et al. 2000). The resulting 740-bp PCR product was digested with BamHI and HindIII and cloned into the pETDuet-1 vector (Novagen) yielding pMS672. HSP22F was overexpressed in *E. coli* BL21 and purified by nickel-nitrilotriacetic acid (Ni–NTA) affinity chromatography according to the manufacturer’s instructions (Qiagen). The region encoding the C-terminal 123 amino acids of CPN60B2 was amplified from cDNA clone AV642726. The resulting 419-bp PCR product was digested with BamHI and HindIII and cloned into the pETDuet-1 vector giving pFW121. The recombinant protein was expressed in *E. coli* ER2566 (NEB) and purified by Ni–NTA affinity chromatography. The hexa-histidine tag was removed by TEV protease cleavage. The region encoding the mature trigger factor (TIG1) protein (lacking the putative N-terminal 64 amino-acid transit peptide) was amplified from cDNA clone AV639812. The 1490-bp PCR product was digested with SapI and NdeI and cloned into pTYB21 (NEB) generating pFW13. TIG1 was expressed in *E. coli* ER2566 and purified via chitin affinity chromatography according to the manufacturer’s instructions (NEB). The region encoding the N-terminal 150 amino acids of HSP70A was amplified from cDNA clone AV642602. The 469-bp PCR product was digested with BamHI and EcoRV and cloned into the pETDuet-1 vector giving pFW76. The HSP70A N-terminus was expressed in *E. coli* ER2566 and purified by Ni–NTA affinity chromatography. 1428 bp of full-length RbcL were amplified from Chlamydomonas DNA, digested with NcoI and EcoRI and cloned into pETDuet-1 giving pFW75. The untagged protein was expressed in *E. coli* ER2566 and purified from inclusion bodies. All proteins were used for the raising of antisera in rabbits. Primers used are listed in Supplementary Table S1.

### Protein analysis and blue-native PAGE

Protein extractions, SDS-PAGE, semi-dry blotting and immunodetections were carried out as described previously (Liu et al. 2005; Schulz-Raffelt et al. 2007). Sample amounts loaded were based on protein determination as described by Lowry et al. (1951) or based on chlorophyll concentrations. Immunodetection was performed using enhanced chemiluminescence (ECL) and the FUSION-FX7 Advance™ imaging system (PEQLAB). The antisera used are against HSP70B and CGE1 (Schroda et al. 2001), CF1β (Lemaire and Wollman 1989), Cyt *f* (Pierre and Popot 1993), and mitochondrial carbonic anhydrase (Agrisera AS11 1737). Densitometric band quantifications after immunodetections were done by the FUSIONCapt Advance program (PEQLAB). Blue-native PAGE was performed with crude membrane and soluble fractions according to published protocols (Schagger et al. 1994; Schagger and von Jagow 1991). Briefly, 5 × 10^7^ cells were harvested by a 2-min centrifugation, washed twice with TMK buffer (10 mM Tris–HCl, pH 6.8, 10 mM MgCl_2_ and 20 mM KCl), and resuspended in 500 µl ACA buffer (750 mM ε-aminocaproic acid, 50 mM Bis-Tris pH 7.0 and 0.5 mM EDTA). The samples were frozen in liquid nitrogen and stored at − 80 °C. Before use, samples were thawed on ice and centrifuged for 15 min at 15,700 g and 4 °C. The supernatants were transferred to new sample tubes and pellets were resuspended in 500 µl ACA buffer. Samples were solubilised with 1% β-DDM and, after centrifugation, supernatants were mixed with loading buffer (750 mM ε-aminocaproic acid and 5% (w/v) Coomassie Brilliant Blue G250). Samples were separated on a 5–15% blue-native polyacrylamide gel.

### Cell fractionations

Isolation of chloroplasts was performed as described previously (Zerges and Rochaix 1998) from heat stressed cw15-302 cells (60 min at 39 °C). Mitochondria were isolated according to Eriksson et al. (1995), but using a BioNebulizer (Glas-Col) for cell disruption. The fractionation of cells into soluble and insoluble/membrane fractions by freezing/thawing was done as described previously (Muranaka et al. 2016).

### Co-immunoprecipitations from soluble cell extracts

Cells were grown at 25 °C or heat-stressed for 1 h at 39 °C. 1 × 10^8^ cells were harvested by centrifugation, washed twice in 20 mM Hepes-KOH, pH 7.2, 80 mM KCl and resuspended in lysis buffer [20 mM Hepes-KOH, pH 7.2, 10 mM KCl, 2.5 mM MgCl_2_, 154 mM NaCl, 0.25 × protease inhibitor cocktail (Roche)] with or without DSP crosslinker (Thermo Scientific) added at a final concentration of 2 mM. Cells were broken by freezing/thawing, incubated for 30 min at 4 °C for crosslinking and centrifuged for 30 min at 4 °C and 18,000 g. Antibody affinity purification, coupling to protein A Sepharose beads and immunoprecipitations were carried out as described previously (Heide et al. 2009). Proteins were eluted from the Sepharose beads by heating for 5–10 min at 65 °C and 2 min at 95 °C in 100 µl of 25 mM NH_4_HCO_3_ and 2% SDS.

### MS sample preparation and analysis

Immunoprecipitated proteins were prepared for nanoLC-MS/MS as described previously (Sommer et al. 2014). MS analysis was performed on a high resolution LC-MS system (Eksigent nanoLC425 coupled to a Triple-TOF 5600+, Sciex) in information dependent acquisition (IDA) mode. HPLC separation was performed in trap-elution mode using a Symmetry C18 column (5 µm particle, 0.18 × 20 mm, Waters) for trapping and a self-packed analytical column (75 µm × 150 mm, 3 µm particle ReproSil-PurC18-AQ, Dr. Maisch) for separation. A constant flow of 300 nl/min was employed and the gradient ramped within 55 min from 2 to 35% of HPLC buffer B (buffer A: 2% acetonitrile, 0.1% formic acid; buffer B: 90% acetonitrile, 0.1% formic acid), followed by washing and equilibration steps. The mass spectrometer was run in IDA mode recording one survey scan (250 ms, 350–1250 m/z) and fragment spectra (200–1800 m/z) of the 45 most intense parent ions (charge state > 2, intensity > 200 cps). Recorded spectra were searched against a reference database including all predicted nuclear-encoded protein sequences of *Chlamydomonas reinhardtii* (JGI v5.5), as well as all mitochondrial and chloroplast proteins (http://chlamycyc.mpimp-golm.mpg.de/files/sequences/protein/). Peptide identification, protein assembly, and label-free quantification was performed using the MaxQuant Software (Version 1.5.3.8) with the “match between runs” option for mass-retention time correlation and an FDR of < 1% for peptides and proteins (Cox et al. 2011). The significance of affected interaction partners was analysed by t-testing with multiple correction [false discovery rate (FDR) ≤ 0.05] by comparing all protein abundances present under continuous light and heat shock conditions. To detect interaction partners enriched in samples from heat-stressed cells, an empirical error probability was calculated and regressed on the abundance of proteins within the heat stress samples. Cubic Hermite spline fit insured monotonicity as a necessary requirement of the probability distribution function. The model for empirical error probability was used to control the error rate (threshold ≤ 0.05) in the statistical assessment of interaction partners according to their enrichment after immunoprecipitation.

### Sequence property calculation and analysis

All 19,609 proteins encoded by the *Chlamydomonas reinhardtii* JGI 5.5 genome sequence were subjected to ChloroP prediction (Emanuelsson et al. 1999) in standard FASTA format. The resulting targeting scores and the predicted transit peptides were used to construct a predicted mature chloroplast proteome comprising 4775 proteins. Sequence properties of predicted mature chloroplast proteins and of HSP22E/F interactors were calculated based on the following amino acid property indices: helicity index (Koehl and Levitt 1999), amphiphilicity index (Cornette et al. 1987), coil index (Ptitsyn and Finkelstein 1983), beta sheet propensity (Crawford et al. 1973) and hydrophibicity index (Fasman 1989). The normalisation uses µ^AAIndex^ and σ^AAIndex^ calculated over random sequences (with n = 5000) using the amino acid frequencies of the whole *Chlamydomonas reinhardtii* proteome. The isoelectric point was calculated according to Kozlowski (2016). Kernel density estimation with a Gaussian kernel was used to visualize the distribution of the individual sequence properties. To compare the sequence property distributions of the HSP22E/F interactome and the chloroplast proteome, a t-test with unequal variance in the log space was performed. Analyses and calculations were performed using the Microsoft F# functional programming language with the bioinformatics library BioFSharp (available on GitHub: https://github.com/CSBiology/BioFSharp) and the graphical library FSharp.Ploty (available on GitHub: https://github.com/muehlhaus/FSharp.Plotly).

### Immunofluorescence staining

Cells were fixed and stained as described previously (Uniacke et al. 2011). Primary antibodies were against HSP22F, RbcL and HSP70A, used in 1:300, 1:8000, and 1:4000 dilutions, respectively. As secondary antibody we used a fluorescein isothiocyanate–labeled goat anti-rabbit antibody (Sigma) in 1:500 dilution. After incubation with the secondary antibody, slides were washed in PBS and a drop of mounting solution containing DAPI (Vectashield, Vector Laboratories) was applied at the center of each slide. Images were obtained with an OLYMPUS BX53 microscope with a violet filter for DAPI and a green filter for FITC using an OLYMPUS DP26 color camera.

### Phylogenetic analyses

Phylogenetic analyses were performed with sequences comprising only the α-crystalline domains (NCBI conserved domain database entry cd06464) of sHsps using the Phylogeny.fr pipeline (Dereeper et al. 2008) implementing algorithms T-Coffee (Notredame et al. 2000), BioNJ (Gascuel 1997), and TreeDyn (Chevenet et al. 2006). The sequences are documented in Supplemental Table S2.

## Results

### The *HSP22E*/*F* genes provide a snapshot of the ongoing diversification of sHsp genes in the *Volvocales*

**Fig. 1:**
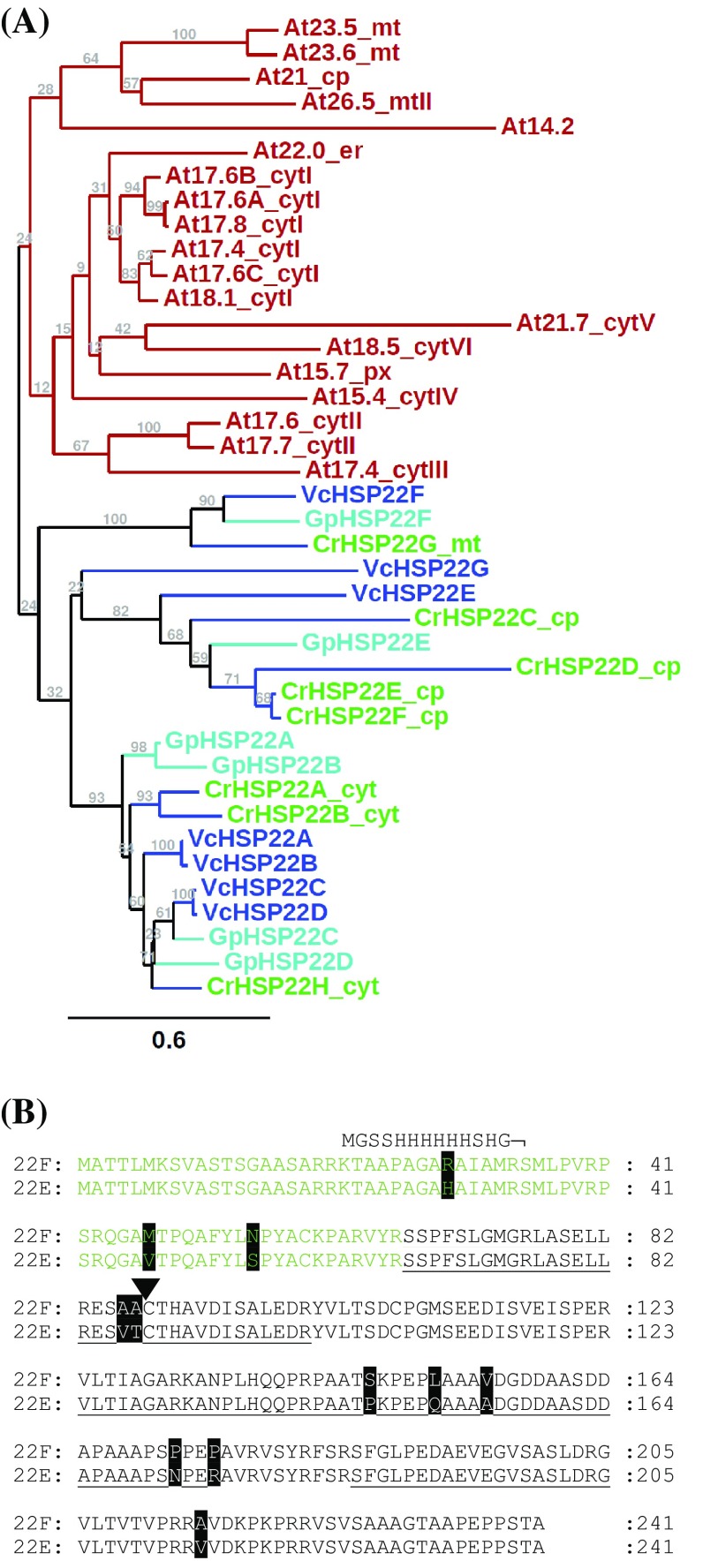
Phylogenetic tree of sHsps and comparison of the Chlamydomonas HSP22E and HSP22F amino acid sequences. **a** Phylogram based on an amino acid sequence alignment of the α-crystalline domains from *Arabidopsis thaliana* (At) and *Volvocales* members *Chlamydomonas reinhardtii* (Cr), *Gonium pectorale* (Gp) and *Volvox carteri* (Vc). Protein names are appended by their predicted intracellular localization (*cyt* cytosol; *cp* chloroplast; *mt* mitochondria; *er* endoplasmic reticulum; *px* peroxisome) and phylogenetic subfamily (roman numbers) as assigned by Waters et al. (2008) and Schroda and Vallon (2009). Support for the branches is given in bootstrap values based on 1000 NJ bootstrap replicates. **b** Alignment of HSP22F and HSP22E protein sequences. Underlined sequences indicate peptides identified by LC-MS/MS analysis of the immunoprecipitated proteins (Supplementary Table S3). Sequences shown in green represent the putative chloroplast transit peptide, sequences in black the putative mature protein. The triangle indicates the cleavage site predicted by ChloroP (Emanuelsson et al. 1999). Differences in both sequences are shaded in black. The sequence on top of that of HSP22F shows where the hexahistidine tag is fused to HSP22F in the recombinant protein

HSP22E and HSP22F are two of eight small heat shock proteins encoded in the Chlamydomonas genome (Merchant et al. 2007; Schroda and Vallon 2009). Like the *HSP22A* and *HSP22B* genes, the *HSP22E* and *HSP22F* genes are oriented head-to-head with ~ 368 bp separating both transcriptional start sites (Supplemental Fig. 1). Nevertheless, each gene contains its own promoter, as judged from the presence of a (degenerated) heat shock element (HSE) and a TATA-box in each. The nucleotide sequences of both genes, including promoter, 5′ untranslated region (UTR), coding region, the single intron, and 16 bp of the 3′ UTR share 93% identity, with most alterations in the promoter region and the 5′ UTR (Supplemental Fig. 2). Hence, the two genes apparently were generated by a recent gene duplication event that comprised sequences just upstream of the HSE to only little after the stop codon. An ongoing expansion of the sHsp gene family in the *Volvocales* is evident also from apparent recent gene duplications in *Volvox carteri*. Here genes *VcHSP22A* and *VcHSP22B* (like *HSP22E* and *HSP22F*) share 98% sequence identity in the coding regions and are located ~ 111 kb apart on the same chromosome. Phylogenetic analyses including all sHsps identified in Arabidopsis, *Chlamydomonas reinhardtii* and two other *Volvocales* members, *Gonium pectorale* and *Volvox carteri*, revealed that Chlamydomonas HSP22E and F form a subfamily together with HSP22C and D, which is clearly separated from a subfamily populated by HSP22A, B, and H and from a third subfamily comprising HSP22G (Fig. 1a). These three *Volvocales* sHsp subfamilies are distinct from the Arabidopsis sHsp subfamilies, indicating that the diversification of sHsp gene families occurred independently in *Volvocales* and land plants from a common ancestor sHsp gene.

### HSP22E and HSP22F are expressed at equal levels and localize to the chloroplast

**Fig. 2:**
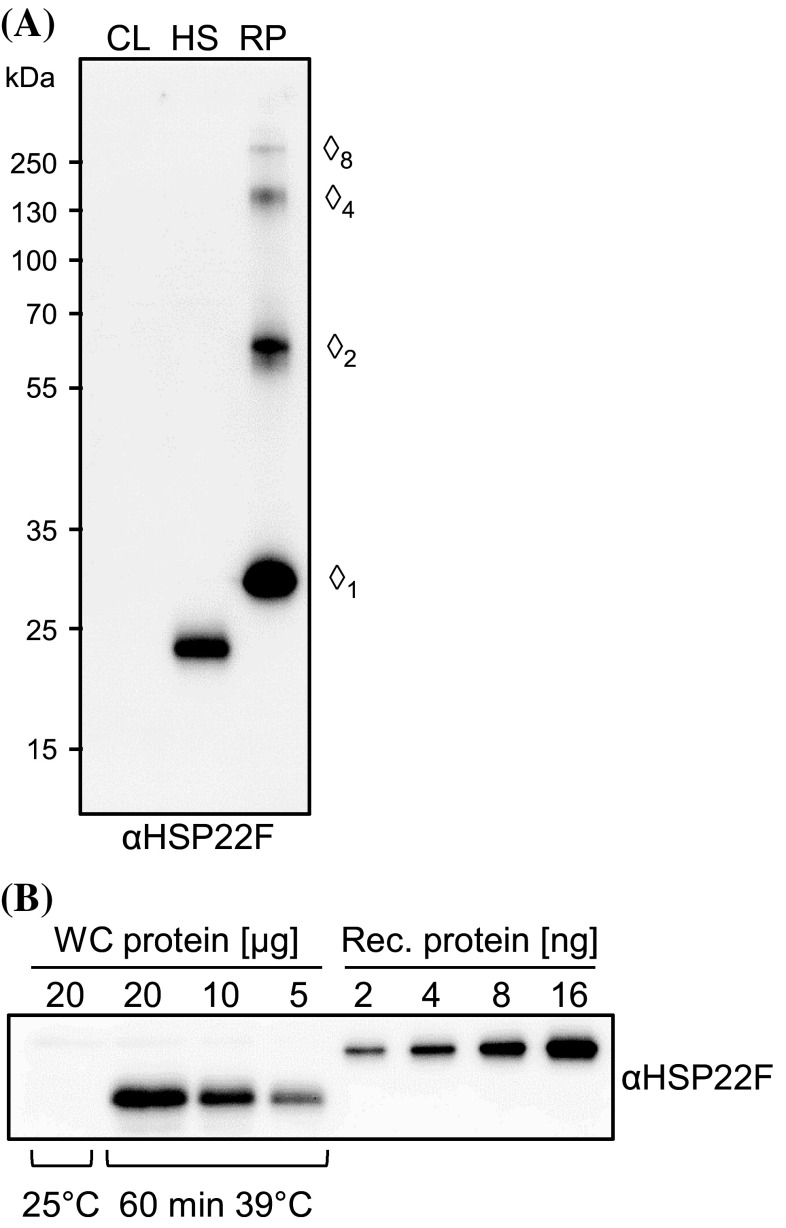
Antibody characterization and analysis of HSP22E/F abundance. **a** Immunodetection of HSP22E/F in total proteins extracted from cw15-302 cells grown at 25 °C (CL) or exposed to 39 °C for 60 min (HS). Proteins loaded corresponded to 1 µg chlorophyll for total cell protein and to 20 ng for recombinant HSP22F protein (RP). Diamonds indicate monomeric recombinant HSP22F and SDS-resistant oligomers with apparent molecular masses of 29 (monomer), 62 (dimer), 139 (tetramer), and 256 kDa (octamer), respectively. **b** 2–16 ng of recombinant HSP22F were separated by SDS-PAGE together with 5–20 µg of whole-cell (WC) proteins from cw15-302 cells exposed to 39 °C for 60 min and immunodetected with the antiserum against HSP22F

**Fig. 3:**
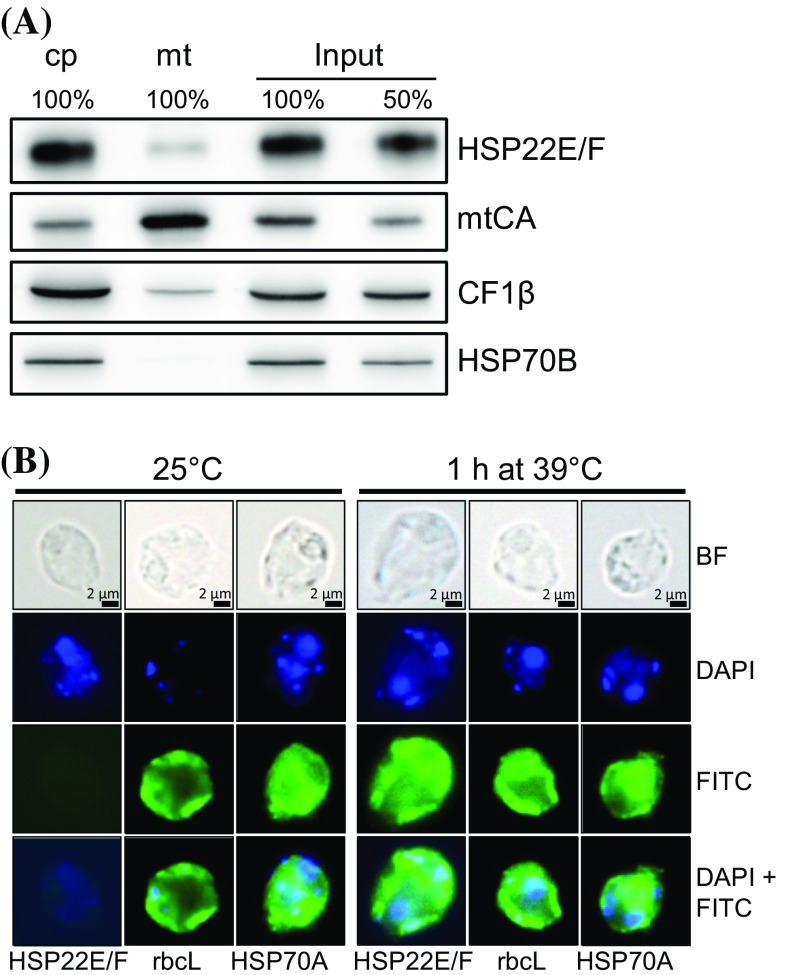
Localization of HSP22E/F to the chloroplast. **a** Subcellular localization of HSP22E/F by immunoblotting. 10 or 3 µg protein (depending on the antiserum used) from whole cells (input), chloroplasts (cp) and mitochondria (mt) isolated from strain cw15-302 exposed to 39 °C for 60 min were separated by SDS-PAGE and immunodetected with antisera against HSP22F, mitochondrial carboanhydrase (mtCA), extrinsic thylakoid membrane protein CF1β, and stromal HSP70B. **b** Microscopy images taken from cells of strain cw15-325 that were kept at 25 °C or exposed to 39 °C for 60 min. Shown are from top to bottom: bright field (BF) images, DAPI staining, immunofluorescence (FITC), and the merge of DAPI and FITC. Antisera used for immunofluorescence were against HSP22F, stromal RbcL, and cytosolic HSP70A

The HSP22E and HSP22F precursors both consist of 241 amino acids with differences only at eleven positions (Fig. 1b). We recombinantly expressed HSP22F with an N-terminal hexahistidine tag replacing part of the presumed chloroplast transit peptide and raised a polyclonal antiserum against this protein. In protein extracts from cells exposed to 39 °C for 60 min the antiserum detected a single protein band with an apparent mass of 22,950 Da on SDS-PAGE, while the recombinant protein had an apparent mass of 29,120 Da (Fig. 2a). nanoLC-MS/MS analyses of immunoprecipitates performed with the HSP22F antiserum on soluble proteins from heat-stressed cells (see below) revealed unique peptides for both HSP22E and HSP22F, suggesting that both proteins populate the band at 22,950 Da. The ratio between ion intensities of peptides ESAACTHAVDISALEDR (HSP22F) and ESVTCTHAVDISALEDR (HSP22E) that can be assumed to ionize with comparable efficiencies, was 1.03 ± 0.05 (n = 4), indicating that both proteins are equally expressed under heat stress conditions (Supplementary Table 1). HSP22E/F levels present in cells exposed to 39 °C for 60 min constituted 0.03% ± 0.004% (n = 3) of total proteins (Fig. 2b). The peptide coverage of the mature HSP22E/F proteins revealed that the chloroplast transit peptide is shorter than predicted by ChloroP (Emanuelsson et al. 1999) (Fig. 1b). When comparing the apparent with the calculated molecular masses (22,950 vs. 18,110 for the mature, and 29,120 vs. 23,229 for the recombinant proteins) it is clear that HSP22E/F have an about 25% larger apparent than calculated molecular mass. The predicted chloroplast localization of HSP22E/F was verified on immunoblots where HSP22E/F (like chloroplast proteins HSP70B and CF1β) were enriched in chloroplasts isolated from heat-stressed cells, while they were depleted in isolated mitochondria (Fig. 3a). Moreover, immunofluorescence analyses revealed a localization of HSP22E/F in the chloroplast of heat-stressed cells, while HSP22E/F were not detectable in cells kept at 25 °C (Fig. 3b).

### HSP22E/F accumulate rapidly upon heat stress and form large complexes with substrate proteins

**Fig. 4:**
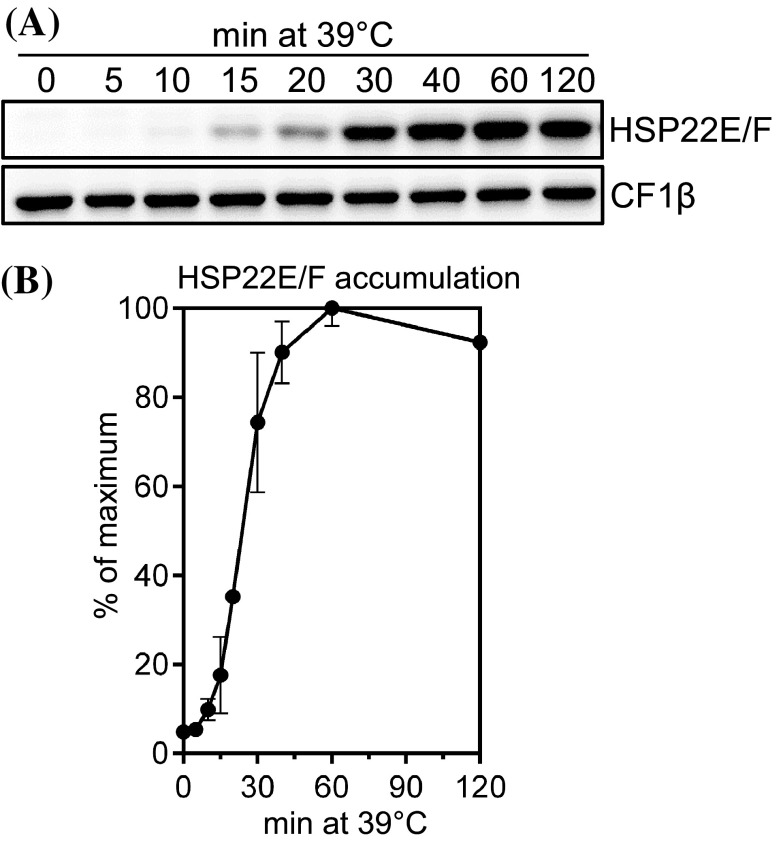
Accumulation of HSP22E/F during heat stress. **a** Immunoblot analysis of HSP22E/F accumulation during heat stress. cw15-302 cells grown at 25 °C were exposed to 39 °C for 120 min. Total proteins corresponding to 0.25 µg chlorophyll from cells harvested at the time points given were separated by SDS-PAGE and analyzed by immunoblotting using antisera against HSP22F and against CF1β as loading control. **b** Quantification of HSP22E/F signal intensities from (**a**). Signals were normalized to the maximal HSP22E/F levels reached during the time course. Error bars represent SD, n = 2

**Fig. 5:**
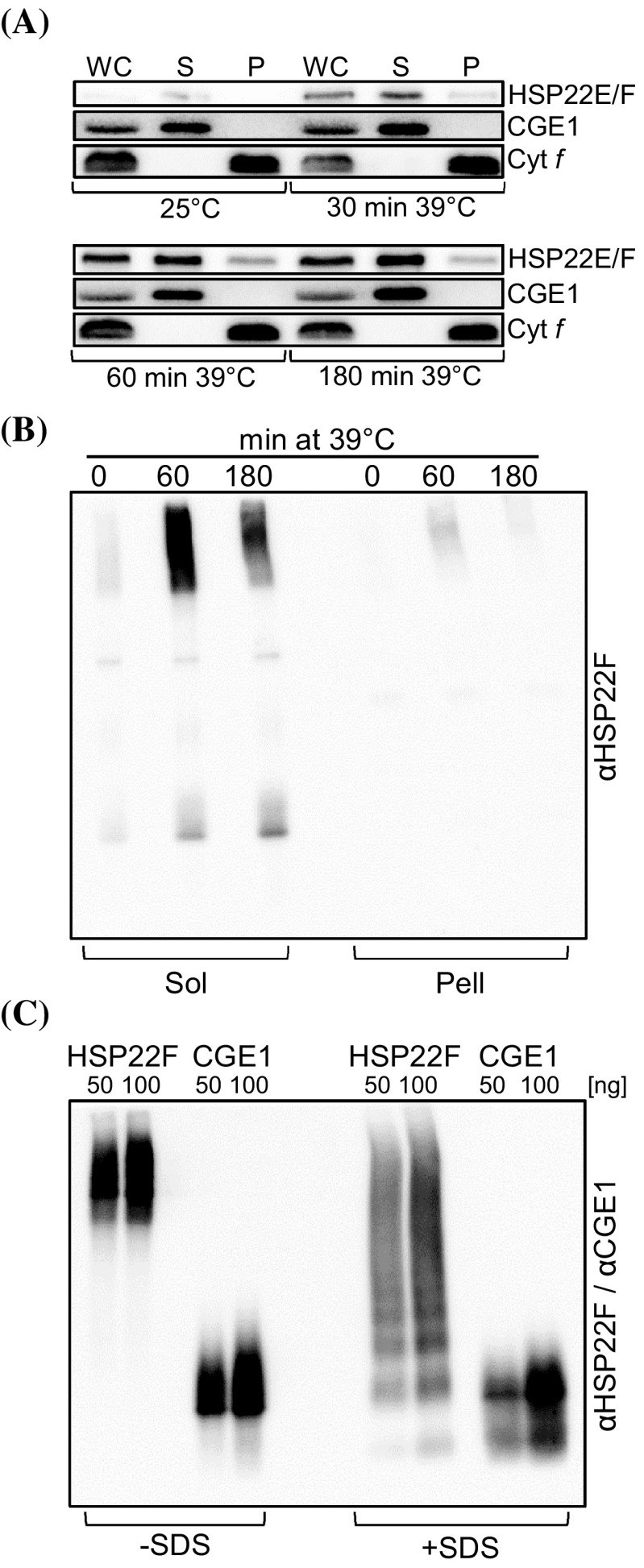
Formation of HSP22E/F high molecular mass complexes during heat stress. **a** Analysis of the partitioning of HSP22E/F to soluble and insoluble/membrane fractions during heat stress. cw15-302 cells grown at 25 °C were exposed to 39 °C for 180 min. Whole cells collected at the time points given (WC) were separated into soluble (S) and insoluble/membrane fractions (P) by two cycles of freezing/thawing. Proteins were separated by SDS-PAGE and immunodetected with antibodies against HSP22F and against integral membrane protein cytochrome *f* (Cyt *f*) or stromal CGE1 as controls. **b** Analysis of HSP22E/F-containing complexes formed during heat stress. cw15-302 cells were exposed to 39 °C for 180 min and cells harvested at the time points given were fractionated into soluble (Sol) and insoluble/membrane (Pell) proteins by freezing-thawing. Protein complexes were separated on a 5–15% blue-native gel and HSP22E/F was detected by immunoblotting. **c** Analysis of oligomers formed by recombinant HSP22F and CGE1. 50 and 100 ng recombinant HSP22F and CGE1 were separated on a 5–15% blue-native gel and detected by immunoblotting. Proteins on the right half of the gel were supplemented with SDS at a final concentration of 2% prior to electrophoresis. The ~ 24-kDa CGE1 protein was used as control as it is known to form stable dimers (Willmund et al. 2007)

To analyze the accumulation kinetics of HSP22E/F during heat stress, HSP22E/F levels were monitored in Chlamydomonas cells exposed to 39 °C for 120 min. The threshold temperature beyond which a heat stress response is triggered in Chlamydomonas has been determined to lie at 37 °C (Rütgers et al. 2017). As shown in Fig. 4, HSP22E/F were present at low levels under non-stress conditions, accumulated to a maximum after 60 min at 39 °C and declined slightly thereafter. Fractionation of heat-stressed cells into soluble proteins and non-soluble proteins/membranes revealed that most of HSP22E/F remained in the soluble fractions during heat stress while only a small part went into the non-soluble/membrane fraction (Fig. 5a). This result was verified in BN-PAGE analyses (Fig. 5b). BN-PAGE also revealed that in cells grown at 25 °C HSP22E/F existed at about equal quantities in low and high molecular mass assemblies in the soluble fraction. In cells exposed to 39 °C for 60 min most of the newly synthesized HSP22E/F proteins accumulated in high molecular mass complexes, whose abundance declined again in cells exposed to heat stress for 180 min. In the non-soluble/membrane fraction HSP22E/F was detected only in complexes of high molecular mass and levels were highest after 60 min of heat stress (Fig. 5b). Recombinant HSP22F formed constitutive oligomers that could be partially disassembled by the addition of SDS (Figs. 2a, 5c).

To identify HSP22E/F substrates we immunoprecipitated HSP22E/F from cells grown at 25 °C and from cells shifted to 39 °C for 60 min (because at this time the abundance of HSP22E/F in large complexes was highest). To remove cell debris, we had to run a pre-clearing centrifugation step prior to immunoprecipitation by which very large protein aggregates most likely were removed. Hence, proteins co-immunoprecipitated with HSP22E/F from the soluble fraction probably existed in smaller complexes/aggregates. Immunoprecipitations were carried out four times for each condition, two in the presence of 2 mM dithiobis[succinimidyl propionate] (DSP) to stabilize transient interactions (Willmund et al. 2008) and two in the absence of the crosslinker. 5% of the immunoprecipitated proteins were used for SDS-PAGE/immunoblotting while the remainder was digested by trypsin and analyzed by nanoLC-MS/MS. In the eight precipitates obtained we identified a total of 168 different proteins (Supplementary Table S3).

**Fig. 6:**
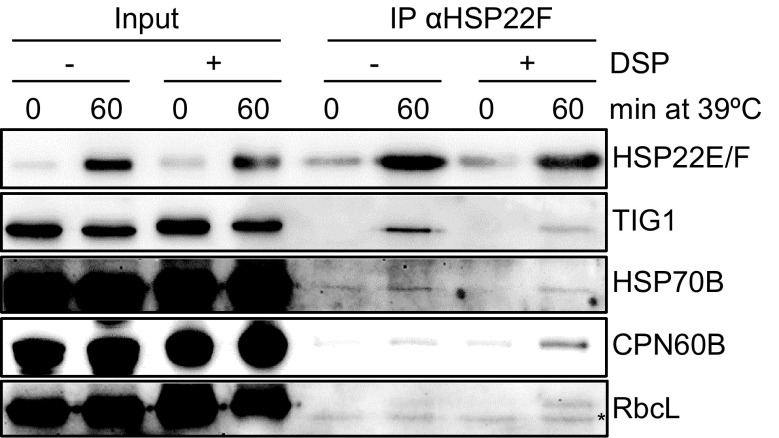
Verification of proteins co-precipitating with HSP22E/F by immunoblot analysis. Total soluble proteins were extracted from cells grown at 25 °C and shifted to 39 °C for 0 or 60 min. Extracts were supplemented with or without the homobifunctional crosslinker DSP prior to the immunoprecipitation of HSP22E/F. 0.3% of the input for immunoprecipitation and 5% of the immunoprecipitates were separated on a 12% SDS–polyacrylamide gel and analyzed by immunoblotting. The asterisk indicates a protein crossreacting with anti-RbcL antibodies

We reasoned that true interaction partners of HSP22E/F should behave similar to their bait, i.e., they should be present at significantly higher amounts in precipitates generated from stressed versus non-stressed cells (Basha et al. 2004). However, for many identified proteins values for peptide intensities were missing in all four replicates done under non-stress conditions (Supplementary Table S3). We therefore developed an algorithm that as training set uses immunoprecipitated proteins for which peptide intensity values were present for both conditions. Those proteins in this training set that have significantly higher ion intensity values under heat stress versus non-stress conditions were defined as true, and those with no significant difference were defined as false interaction partners. All proteins of the training set were then binned according to the ion intensities of peptides derived from proteins precipitated under heat stress conditions. For each intensity bin the fraction of false to true interaction partners was determined to give an empirical error probability, which was plotted against the ion intensities of all peptides recovered under heat stress conditions (Supplemental Fig. S3). Finally, a curve was fitted and the resulting function allowed assigning an empirical error probability to every immunoprecipitated protein based on the ion intensity of the peptides recovered under heat stress conditions. 39 of the 168 co-precipitated proteins had a p score ≤ 0.05 and were therefore classified as proteins that interact with high confidence directly or indirectly with HSP22E/F under heat stress (Table 1). To confirm some of them, we raised antisera against chloroplast trigger factor (TIG1), chaperone CPN60B and the Rubisco large subunit (rbcL), and used an antiserum available against chaperone HSP70B to test for the presence of these proteins in HSP22E/F immunoprecipitates by immunoblotting. As shown in Fig. 6, all these proteins were found to be enriched in HSP22E/F immunoprecipitates generated from heat-stressed cells.

**Table 1:** Proteins directly or indirectly interacting with HSP22E/F under heat stress

Gene ID	Name	Description	Loc	Rank CL	Rank HS	Function	PScore
Cre14.g617450	HSP22E	Heat shock protein 22E	cp^c^	973	82	Molecular chaperone	0.001
Cre14.g617400	HSP22F	Heat shock protein 22F	cp^c^	973	82	Molecular chaperone	0.001
Cre01.g001750	TIG1	Chloroplast trigger factor	cp^c^	1085	1159	Molecular chaperone	0.018
Cre04.g231222	CPN60A	Chaperonin 60A	cp^a^	nd	nd	Molecular chaperone	0.021
Cre07.g339150	CPN60B2	Chaperonin 60B2	cp^a^	157	17	Molecular chaperone	0.025
Cre06.g250100	HSP70B	Heat shock protein 70B	cp^a^	114	19	Molecular chaperone	0.045
Cre07.g318800	HSP22A	Heat shock protein 22A	cyt^a^	nd	7	Molecular chaperone	0.014
Cre08.g372100	HSP70A	Heat shock protein 70A	cyt^a^	60	11	Molecular chaperone	0.032
Cre01.g056331	DJ1L	Homolog of human DJ-1 like/bacterial YajL	cp^b^	nd	nd	ROS protection	0.047
Cre13.g562150	YCHFL1	GTP-binding protein-related	cp^b^	808	nd	ROS signaling	0.018
Cre12.g530650	GLN2	Glutamine synthetase	cp^a^	68	41	N-metabolism	0.001
Cre06.g308500	CMPS1	Carbamoyl phosphate synthase, small subunit	cp^a^	512	620	Arginine/Pyrimidin biosynthesis	0.049
Cre03.g203850	ATS1	ATP-sulfurylase	cp^a^	267	274	S-metabolism	0.033
Cre09.g387800	FER1	Pre-apoferritin	cp^a^	277	436	Fe-metabolism	0.032
Cre04.g214150	THI4	Thiazole biosynthetic enzyme	cp^b^	155	624	Vitamin biosynthesis	0.003
Cre02.g085450	CPX1	Coproporphyrinogen III oxidase	cp^a^	404	599	Chlorophyll biosynthesis	0.018
chlL	chlL	Protochlorophyllide reductase subunit L	cp^a^	nd	nd	Chlorophyll biosynthesis	0.018
chlB	chlB	Protochlorophyllide reductase subunit B	cp^a^	600	nd	Chlorophyll biosynthesis	0.037
chlN	chlN	Protochlorophyllide reductase subunit N	cp^a^	602	nd	Chlorophyll biosynthesis	0.039
Cre01.g050950	GGR1	Geranylgeranyl reductase	cp^a^	949	1037	Chlorophyll biosynthesis	0.018
Cre03.g175400	PGI1	Phosphoglucose isomerase	cp^b^	293	358	Sugar metabolism	0.017
Cre03.g185250	SSS2	Soluble starch synthase II	cp^a^	nd	nd	Starch synthesis	0.038
Cre06.g282000	SSS3	Soluble starch synthase III	cp^a^	878	796	Starch synthesis	0.018
Cre10.g444700	SBE3	Starch branching enzyme	cp^a^	521	801	Starch synthesis	0.017
Cre08.g373450	SBE4	Starch branching enzyme	cp^a^	nd	nd	Starch synthesis	0.050
rbcL	rbcL	Rubisco large subunit	cp^a^	2	10	Calvin cycle	0.023
Cre04.g229300	RCA1	Rubisco activase	cp^a^	248	135	Calvin cycle	0.042
Cre12.g509650	PDS1	Phytoene desaturase	cp^a^	nd	nd	Carotenoid biosynthesis	0.018
Cre04.g231026	SRP43	Chloroplast signal recognition particle subunit	cp^a^	nd	nd	Thylakoid targeting	0.019
Cre01.g020918	PREP1	Presequence protease 1	cp^a^	nd	nd	Cp transit peptide degradation	0.039
atpA	atpA	ATP synthase CF1 alpha subunit	cp^a^	21	61	ATP synthase	0.042
Cre03.g156600	PGR7	Proton gradient regulation 7	cp^a^	nd	nd	Thylakoidal electron transport	0.041
Cre17.g702500	TAB2	PsaB RNA binding protein	cp^a^	nd	nd	PSI biogenesis	0.048
Cre16.g659950	PRPS5	Plastid ribosomal protein S5	cp^a^	364	434	Ribosome	0.043
Cre02.g141400	PCK1	Phosphoenolpyruvate carboxykinase	cyt/cp^b^	29	779	Gluconeogenesis	0.020
Cre02.g099850	PDC2	Pyruvate dehydrogenase, E1 component, alpha subunit	cp^a^	354	369	Acetyl-CoA synthesis	0.043
Cre02.g085900	IMPL1	Myo-inositol monophosphatase like 1	cp^a^	nd	769	Signal transduction	0.027
Cre16.g653350	–	UDP-3-*O*-acyl *N*-acetylglycosamine deacetylase	cp^b^	801	1062	Lipid A biosynthesis	0.034
Cre16.g671950	–	Putative nuclease containing GIY-YIG domain	cp^b^	702	1157	DNA cleavage	0.004
Cre15.g644051	–	Protein with P-loop nucleoside triphosphate hydrolase	cp^b^	453	1153	Unknown	0.018
Cre02.g073550	–	Putative nucleosome assembly protein	cyt/cp^b^	268	349	Unknown	0.045

Intracellular localization (Loc)

^a^Based on literature reports on the Chlamydomonas proteins or their orthologs in land plants

^b^Based on ChloroP and TargetP predictions of Volvocacean and plant proteins, respectively

^c^Based on our own experimental evidence (see Supplementary Table S3 for details). Ranks among 1207 quantified soluble proteins from non-stressed Chlamydomonas cells (CL) and cells exposed to heat stress for 24 h (HS) were taken from Schroda et al. (2015)

## Discussion

### Recent gene duplications indicate an ongoing diversification of sHsp genes in the *Volvocales*

Phylogenetic analyses on the amino acid sequences of sHsps from *Arabidopsis thaliana* and three members of the *Volvocales* (Fig. 1a) confirm previous assignments of the 19 Arabidopsis sHsps to subfamilies containing members targeted to chloroplasts/mitochondria, ER, cytosol, and peroxisomes (Waters et al. 2008; Waters and Rioflorido 2007; Waters and Vierling 1999). These analyses also confirm that the diversification of the sHsps in plants has occurred after the divergence of the ancestor of land plants and the *Volvocales* (Waters and Vierling 1999). The apparent recent duplication of the Chlamydomonas *HSP22E*/*F* and the Volvox *HSP22A*/*B* genes indicates that the diversification of sHsp genes in the *Volvocales* is ongoing, as is true for land plants (Waters et al. 2008). The presence of more sHsp genes in land plant species (19 in Arabidopsis, 23 in rice and 39 in poplar) than in *Volvocales* species (8 in *Chlamydomonas reinhardtii*, 7 in *Volvox carteri*, and 6 in *Gonium pectorale*) however suggests that there is less evolutionary pressure on diversification in the *Volvocales* as compared with land plants (Waters et al. 2008). Phylogenetic analyses on the *Volvocales* sequences allow separating three subfamilies that based on the positions of the Chlamydomonas sequences can be termed ABH, CDEF, and G (Fig. 1a). Experimental evidence on the localization of HSP22A to the cytosol (Eisenberg-Domovich et al. 1994) and of HSP22E/F to the chloroplast (Fig. 3) in addition to previous predictions (Schroda and Vallon 2009) suggests that members of the ABH, CDEF, and G subfamilies might be targeted to cytosol, chloroplast and mitochondria, respectively. However, this notion awaits experimental testing. While in previous studies HSP22A, B, C, and E/F have been detected by mass spectrometry in heat-stressed Chlamydomonas cells, HSP22D, G, and H have not been detected (Hemme et al. 2014; Mühlhaus et al. 2011), which is in line with RNA-seq data (Phytozome v12.1). Therefore, HSP22D, G, and H might be expressed only under specific stress conditions, only during the sexual cycle, or might not be expressed at all.

### HSP22E/F are in a dynamic equilibrium between larger oligomers and smaller assembly states in vivo

In non-stressed cells, HSP22E/F were found in large complexes as well as in smaller assemblies, while recombinant HSP22F was found exclusively in large oligomers (Fig. 5b, c). This difference might be due to the high concentration of the pure recombinant protein in vitro when compared with the more diluted native protein that is in company of many other chloroplast proteins and solutes in vivo (Haslbeck et al. 2004). Hence, like other sHsps (Bova et al. 2002; Fleckenstein et al. 2015; Haslbeck et al. 2004), also HSP22E/F appear to be in a dynamic equilibrium between larger oligomers and smaller assembly states in vivo. HSP22E/F synthesized de novo in heat-stressed cells accumulated largely in high molecular mass complexes that, according to the immunoprecipitation results, contained thermolabile substrate proteins (Figs. 5b, 6). Our data do not allow judging whether HSP22E/F in these high molecular mass complexes exist as dissociated monomers/dimers that intercalate into aggregated substrate proteins, or as an oligomeric core to which unfolded proteins attach. Both situations have been proposed previously for yeast Hsp26 and Hsp42, respectively (Haslbeck et al. 2004).

### 39 high-confidence HSP22E/F interactors

Using a differential immunoprecipitation strategy and a stringent filtering algorithm, we could extract 39 high-confidence HSP22E/F interactors from a total of 168 proteins identified in eight immunoprecipitates (Table 1; Supplementary Table S3). That these 39 proteins truly interact (directly or indirectly) with HSP22E/F is supported by four lines of evidence: first, 35 of the 39 proteins are very likely targeted to the chloroplast, as judged from literature reports and from the presence of N-terminal extensions predicted to qualify as chloroplast transit peptides (Table 1; Supplementary Table S3). Two proteins, HSP22A and HSP70A, are clearly localized to the cytosol and presumably were precipitated because HSP22E/F-containing chloroplast aggregates fused with HSP22A- and HSP70A-containing cytosolic aggregates upon the mixing of compartments during cell lysis, or because the antiserum raised against HSP22F cross-reacted with HSP22A. Second, the identified proteins cover a wide range of abundance classes with rbcL and trigger factor (TIG1) as representatives for high abundance proteins (rank 2) and low abundance proteins (rank 1085), respectively (Table 1). Moreover, although cellular levels of 18 of the 39 high-confident HSP22E/F interactors have been shown to decrease during heat stress (Hemme et al. 2014; Mühlhaus et al. 2011), they were enriched by immunoprecipitation with anti-HSP22E/F antibodies in heat-stressed cells. Third, with antisera against TIG1, HSP70B, CPN60B, and rbcL we could verify that these proteins were enriched in anti-HSP22E/F precipitates from heat-stressed versus non-stressed cells (Fig. 6). Fourth, homologs of HSP22E/F interactors PEP carboxykinase, ribosomal protein S5, rbcL, HSP70B, TIG1 and atpA, have also been found to interact with sHsps in *E. coli, Synechocystis*, yeast, *D. radiodurans, C. elegans*, or maize chloroplasts (Basha et al. 2004; Bepperling et al. 2012; Fleckenstein et al. 2015; Fu et al. 2013; Haslbeck et al. 2004; Hu et al. 2015).

### HSP22E/F interactors in heat-stressed cells provide insights into cellular processes affected by heat

The proteins identified as interactors of sHsps in these organisms and of HSP22E/F in the chloroplast have in common that they cover a wide range of functions including metabolism, translation, signal transduction and folding (Table 1; Supplementary Table S3). Of the proteins involved in folding it can be assumed that HSP70B and CPN60A/B do not directly interact with HSP22E/F, but are associated with denatured proteins in HSP22E/F-containing aggregates to support their refolding to the native state. Accordingly, the homologous GroEL system from *E.coli* has been shown to support the DnaK-dependent refolding of some sHsp substrates (Mogk et al. 2003a). In contrast to DnaK and GroEL, trigger factor was previously identified as a direct sHsp target in *E.coli* (Fu et al. 2013), therefore indicating that trigger factor is a thermolabile protein and that this is likely true also for its ortholog TIG1 in the chloroplast. For the chloroplast Yaj1 homolog it is not clear whether it is itself thermolabile or is associated with aggregates via its chaperone activity in order to repair proteins that aggregated because they got glycated during heat stress (Richarme et al. 2015).

**Fig. 7:**
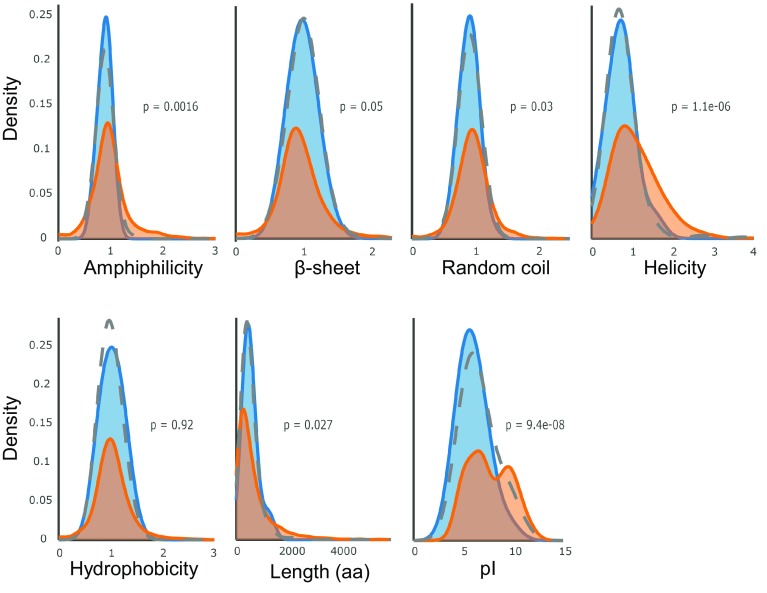
Amino acid sequence property distributions. Kernel density estimation shows the comparison between different sequence property distributions of the 34 high-confidence HSP22E/F interactors excluding sHsps, Hsp70s and Cpn60s (blue) and the 4775 mature chloroplast proteins predicted by ChloroP (orange). The grey dotted line indicates the distribution of all 160 proteins identified in the HSP22E/F immunoprecipitate, excluding sHsps, Hsp70s and Cpn60s

All other proteins identified in the HSP22E/F immunoprecipitates can be assumed to directly interact with HSP22E/F. To investigate whether this interaction is favored by a certain structural property, we tested whether the putative HSP22E/F substrates (proteins co-immunoprecipitated with HSP22E/F excluding sHsps, Hsp70s and Cpn60s) differed from the predicted chloroplast proteome regarding hydrophobicity, amphiphilicity, length, pI, or content of β-sheets, random coils and α-helices. As shown in Fig. 7, the property distributions of the HSP22E/F substrates differed from the predicted chloroplast proteome by a lower helicity and lower isoelectric point (pI) (p < 0.001). Interestingly, a bias for substrates with acidic pI has recently been observed for the Sip1 and Hsp16.2 sHsps from *C. elegans* (Fleckenstein et al. 2015) and therefore an acidic pI might represent a conserved property of sHsp substrates.

Another common property of HSP22E/F substrate proteins in heat-stressed cells is their thermolability. What may cause a protein to be thermolabile? A protein might be thermolabile because its optimal function at the organism’s standard growth temperature requires a delicate construction. Alternatively, mutations accumulating in a protein with the prospect for a novel trait may render it a metastable Hsp90 substrate that would unfold when Hsp90 is sequestered to unfolding proteins during heat stress (Lindquist 2009). While in both cases thermolability is unavoidable, it should be a conserved trait only in the former case. Accordingly, candidate proteins apparently exhibiting thermolability also in other organisms are trigger factor (Fu et al. 2013), glutamine synthetase (Castro-Rodriguez et al. 2015), PEP carboxykinase (Bepperling et al. 2012; Fu et al. 2013; Hu et al. 2015), Rubisco activase (Feller et al. 1998), the α- and β-subunits of the ATP synthase (Basha et al. 2004; Fu et al. 2013; Hu et al. 2015), rbcL (Hu et al. 2015) and the ribosomal protein S5 (Bepperling et al. 2012; Fu et al. 2013). Also phytoene desaturase might be a highly unstable protein, as it was proposed to require Hsp21 in tomato chloroplasts for optimal activity (Neta-Sharir et al. 2005).

However, thermolability of a protein might also be a desired trait, for example as part of a mechanism enabling an organism to rapidly react to thermal stress. This might generally hold for energy-requiring anabolic reactions like chlorophyll biosynthesis, CO_2_-fixation, starch synthesis, sulfur and nitrogen fixation, or protein biosynthesis (Table 1; Supplementary Table S3). The rapid gearing down of these pathways upon heat stress—because key enzymes are constructed thermolabile—would make ATP and reducing equivalents available e.g. for the de novo synthesis and fueling of molecular chaperones, or for the synthesis of saturated fatty acids in order to restore membrane viscosity, as proposed previously (Hemme et al. 2014; Schroda et al. 2015). Also here, thermolability of a protein should be a conserved trait, as apparently is true for glutamine synthetase, PEP carboxykinase, Rubisco activase, Rubisco, and ribosomal protein S5. Compounds accumulating as a consequence of the heat-induced inactivation of an enzyme constructed to be thermolabile may also serve as signaling molecules to trigger protective responses. For example, if Chlamydomonas IMPL1 (Table 1), like its homolog in Arabidopsis chloroplasts, was involved in the recycling of myo-inositol from inositol phosphate second messengers (Nourbakhsh et al. 2015), the longer life-times of the latter could potentially enhance intracellular signaling cascades. Finally, also the thermal inactivation of a repressor protein may unleash responses that enable an organism to trigger heat stress response programs. This might, for example, be the case for the Chlamydomonas YchF homolog YCHFL1 (Table 1). *E. coli* YchF has been shown to bind and inactivate H_2_O_2_-detoxifying enzymes under non-stress conditions (Hannemann et al. 2016). Oxidative stress has been shown to inactivate YchF and to alleviate the inhibition of antioxidant enzymes. Therefore, heat inactivation of the thermolabile chloroplast YchF homolog might lead to the activation of antioxidant enzymes during heat stress.

## Electronic supplementary material

Below is the link to the electronic supplementary material.

Supplementary material 1 (PPTX 52 KB)

Supplementary material 2 (PPTX 67 KB)

Supplementary material 3 (PPTX 69 KB)

Supplementary material 4 (DOCX 15 KB)

Supplementary material 5 (DOCX 21 KB)

Supplementary material 6 (XLSX 175 KB)
