# Supplementary material for: Substrates of the chloroplast small heat shock proteins 22E/F point to thermolability as a regulative switch for heat acclimation in Chlamydomonas reinhardtii
Source: Plant Mol Biol. 2017 Nov 1;95(6):579–91. doi: 10.1007/s11103-017-0672-y (PMC5700999; doi:10.1007/s11103-017-0672-y)
Supplement: Supplementary file 1 — Supplementary material 1 (PPTX 52 KB) [file 11103_2017_672_MOESM1_ESM.pptx]

## Slide 1
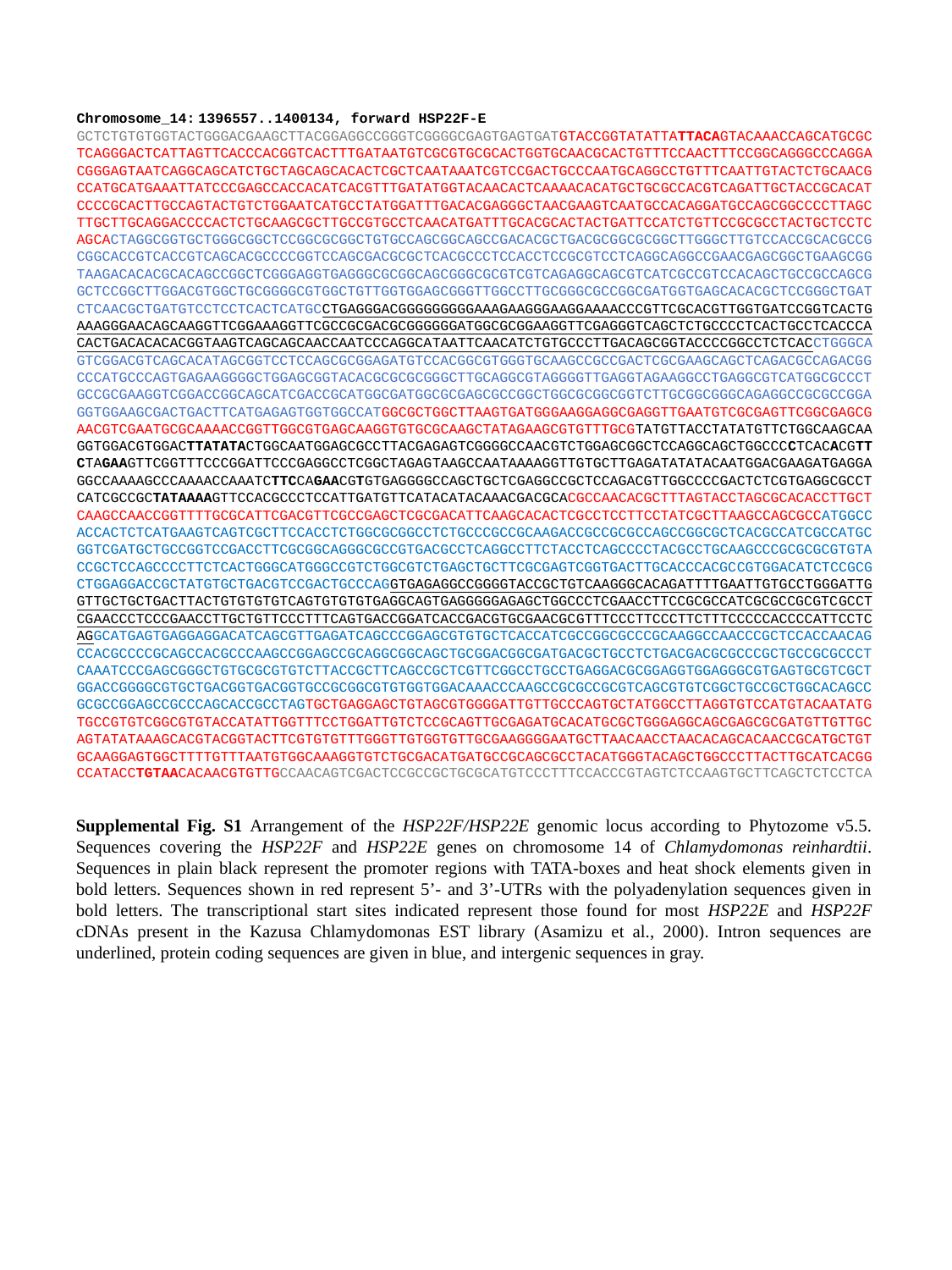

Supplemental Fig. S1 Arrangement of the HSP22F/HSP22E genomic locus according to Phytozome v5.5. Sequences covering the HSP22F and HSP22E genes on chromosome 14 of Chlamydomonas reinhardtii. Sequences in plain black represent the promoter regions with TATA-boxes and heat shock elements given in bold letters. Sequences shown in red represent 5’- and 3’-UTRs with the polyadenylation sequences given in bold letters. The transcriptional start sites indicated represent those found for most HSP22E and HSP22F cDNAs present in the Kazusa Chlamydomonas EST library (Asamizu et al., 2000). Intron sequences are underlined, protein coding sequences are given in blue, and intergenic sequences in gray.
