# Supplementary material for: Substrates of the chloroplast small heat shock proteins 22E/F point to thermolability as a regulative switch for heat acclimation in Chlamydomonas reinhardtii
Source: Plant Mol Biol. 2017 Nov 1;95(6):579–91. doi: 10.1007/s11103-017-0672-y (PMC5700999; doi:10.1007/s11103-017-0672-y)
Supplement: Supplementary file 2 — Supplementary material 2 (PPTX 67 KB) [file 11103_2017_672_MOESM2_ESM.pptx]

## Slide 1
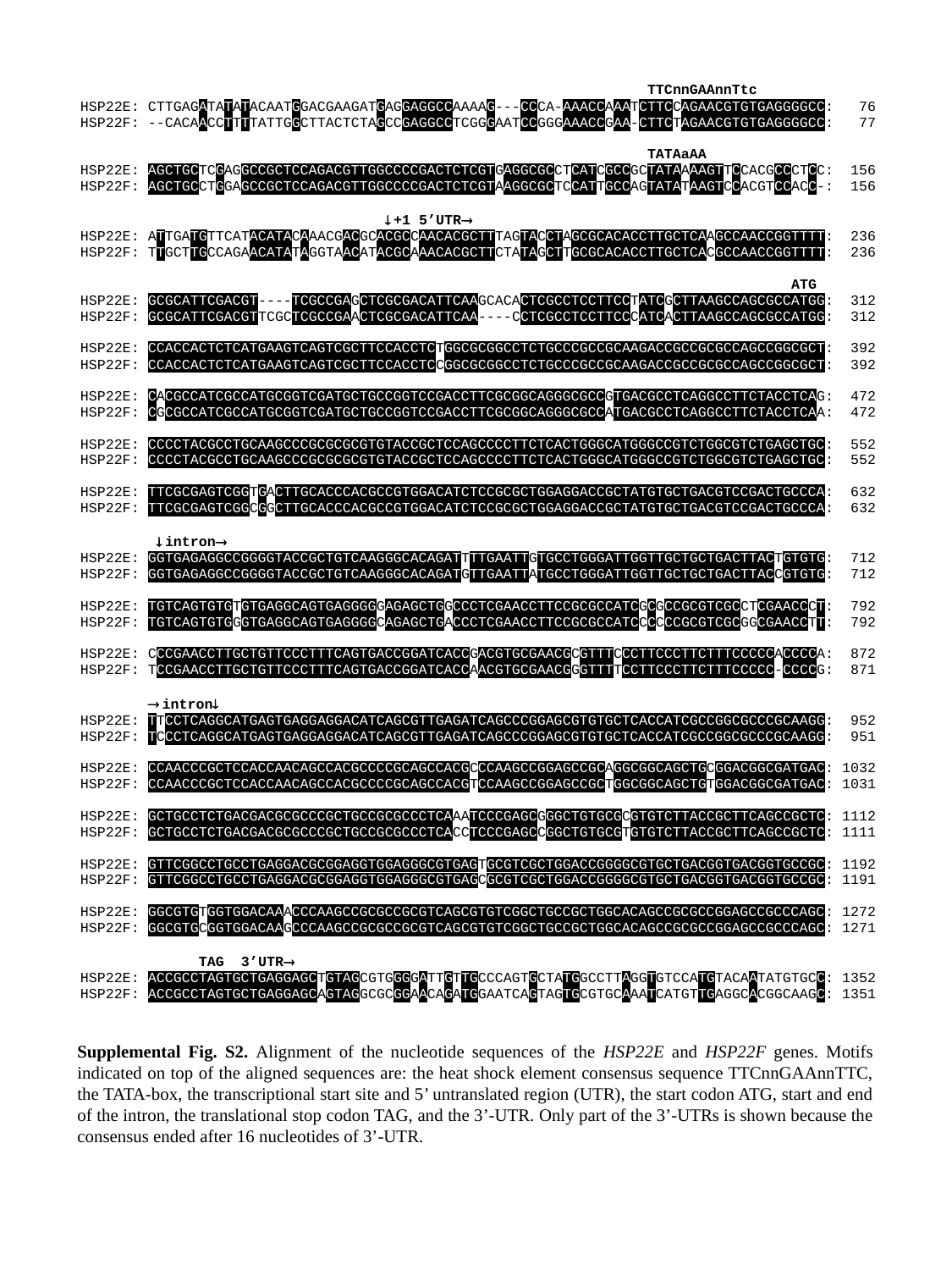

Supplemental Fig. S2. Alignment of the nucleotide sequences of the HSP22E and HSP22F genes. Motifs indicated on top of the aligned sequences are: the heat shock element consensus sequence TTCnnGAAnnTTC, the TATA-box, the transcriptional start site and 5’ untranslated region (UTR), the start codon ATG, start and end of the intron, the translational stop codon TAG, and the 3’-UTR. Only part of the 3’-UTRs is shown because the consensus ended after 16 nucleotides of 3’-UTR.
