# Supplementary material for: Substrates of the chloroplast small heat shock proteins 22E/F point to thermolability as a regulative switch for heat acclimation in Chlamydomonas reinhardtii
Source: Plant Mol Biol. 2017 Nov 1;95(6):579–91. doi: 10.1007/s11103-017-0672-y (PMC5700999; doi:10.1007/s11103-017-0672-y)
Supplement: Supplementary file 3 — Supplementary material 3 (PPTX 69 KB) [file 11103_2017_672_MOESM3_ESM.pptx]

## Slide 1
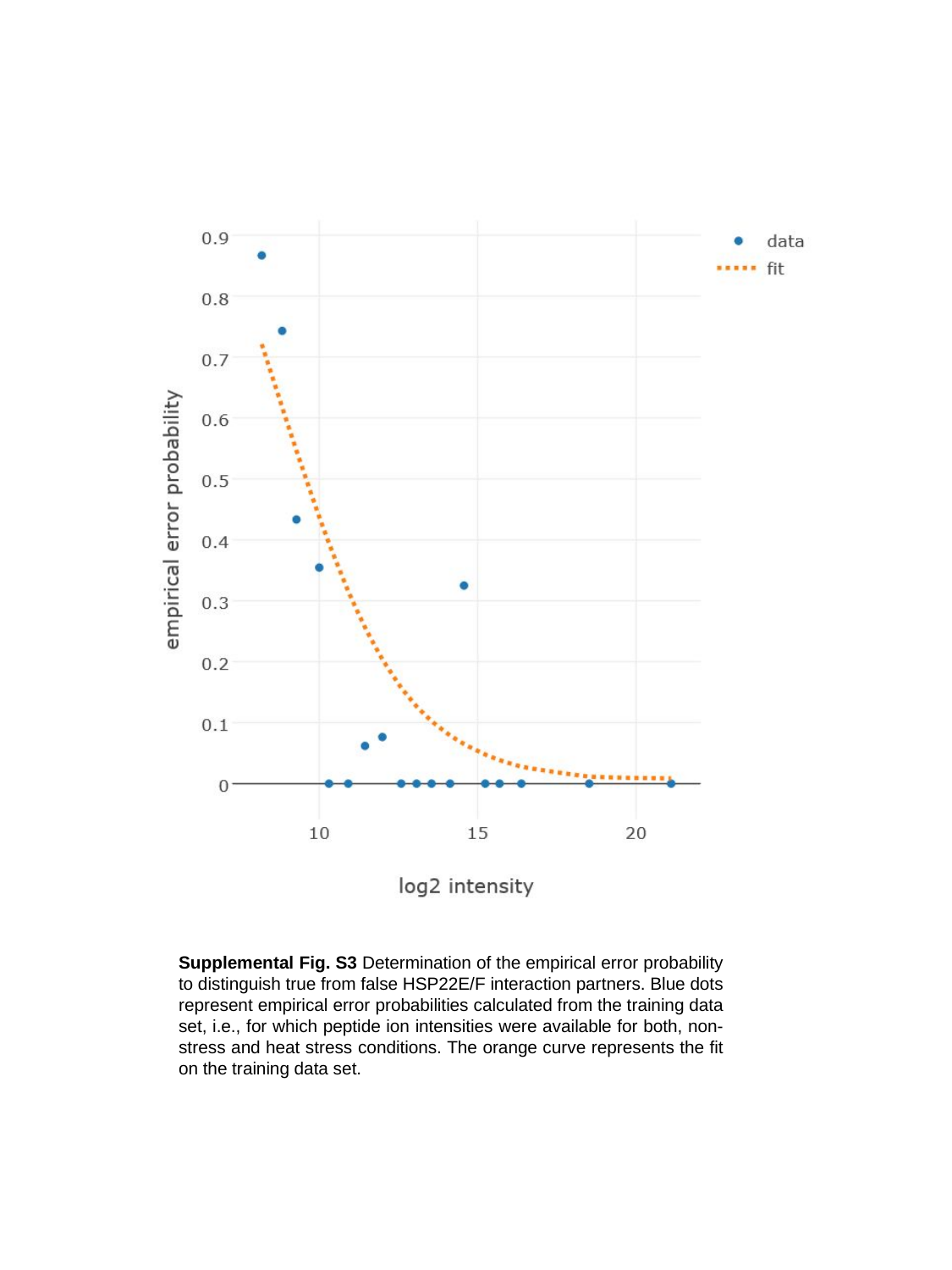

Supplemental Fig. S3 Determination of the empirical error probability to distinguish true from false HSP22E/F interaction partners. Blue dots represent empirical error probabilities calculated from the training data set, i.e., for which peptide ion intensities were available for both, non-stress and heat stress conditions. The orange curve represents the fit on the training data set.
