# Supplementary material for: Substrates of the chloroplast small heat shock proteins 22E/F point to thermolability as a regulative switch for heat acclimation in Chlamydomonas reinhardtii
Source: Plant Mol Biol. 2017 Nov 1;95(6):579–91. doi: 10.1007/s11103-017-0672-y (PMC5700999; doi:10.1007/s11103-017-0672-y)
Supplement: Supplementary file 4 — Supplementary material 4 (DOCX 15 KB) [file 11103_2017_672_MOESM4_ESM.docx]

**Supplementary Table S1.** Primers used for clonings**.**

| **Gene** | **Forward** | **Reverse** |
| --- | --- | --- |
| HSP22F | 5‘-CATTGGGATCCATGCTGCCGGTCCGACCTTCGC-3’ | 5‘-GCCCCTAAGCTTGCTTGCAGGACCCCACTC-3‘ |
| CPN60B2 | 5´- GCCAGGATCCGGAGAATTTATACTTCCAGGGTCAGAAGGTGGACTCCATC-3´ | 5´- CGCGCGAAGCTTTTAGTAGTCGTAGTCACCGC-3´ |
| TIG1 | 5´-GGTGGTTGCTCTTCCAACGCTGCTCAGGTCTGTGCTGCTGCTGG-3´ | 5´-GGTGGCATATGTTACACCGCCACGGGTTCCC-3´ |
| HSP70A | 5´-CAGTAGGATCCGGCAAGGAGGCCCCCGCTA-3´ | 5‘-AAGTAGGATATCACGGTCACCACGGCCTTC-3´ |
| RbcL | 5´-GGGCGCCATGGTTCCACAAACAGAAAC-3´ | 5´- GCGCCGAATTCTTAAAGTTTGTCAATAGTATCAAAT-3´ |
