# Supplementary material for: Substrates of the chloroplast small heat shock proteins 22E/F point to thermolability as a regulative switch for heat acclimation in Chlamydomonas reinhardtii
Source: Plant Mol Biol. 2017 Nov 1;95(6):579–91. doi: 10.1007/s11103-017-0672-y (PMC5700999; doi:10.1007/s11103-017-0672-y)
Supplement: Supplementary file 5 — Supplementary material 5 (DOCX 21 KB) [file 11103_2017_672_MOESM5_ESM.docx]

**Supplementary Table S2.** List of sHSP sequences from *Chlamydomonas reinhardtii* (Cr), *Volvox carteri* (Vc), *Gonium pectorale* (Gp), and *Arabidopsis thaliana* (At) used for phylogenetic analyses. Given are the (assigned) common name and accession numbers from Phytozome and/or GeneBank (if an entry is missing the deposited sequence was incomplete or absent). Sequences in red depict the alpha-crystalline domains according to the NCBI conserved domain database entry cd06464. Protein names are appended by their predicted/reported intracellular localization: cyt – cytosol; cp – chloroplast; mt – mitochondria; er – endoplasmic reticulum; px – peroxisome. Roman numbers indicate the phylogenetic subfamily assigned by Waters et al. (2008).

>CrHSP22A_cyt / Cre07.g318800 / gi|158281514|gb|EDP07269|

MALSNYVFGNSAADPFFTEMDRAVNRMINNALGVAPTSAGKAGHTHAPMDIIESPTAFELHADAPGMGPDDVKVELQEGVLMVTGERKLSHTTKEAGGKVWRSERTAYSFSRAFSLPENANPDGITAAMDKGVLVVTVPKREPPAKPEPKRIAVTGA

>CrHSP22B / gi|158281329|gb|EDP07084|

MALISLFSEPIFDAELTPLGLGFASEDAFQHGHERQRKRHTERHTHERDNIATPRDQSWDLSGPTAPMDIVETPSGYELHADAPGLGPRDVKVELHNGVLQISGSRKLHHESKDLRGRLLRRERTAYSFSRAFSLPENANPDGITAAMDKGVLVVTVPKRPHATAGPPAPKRIAVAAAPAAKL

>CrHSP22C / Cre03.g145787 / gi|158275728|gb|EDP01504|

MSFNHALRRSASQLLARAAAANTAAELPTMAMGTVRNLSSRSAGDLVLGPFRGCSPRYRSLAPFLQPSSLGSLSRAMDELLQMDRHLTAELANVFGDSPLARNSPLFSDSPFRSATSKSVSVTRPTQFRAFEDRYELQADMPGVAESDLHIELDAEEGLLTISGARRDGASPPIPPAPATAASAGPAATASAATGTQQQPASAPATASAAADEADAAAAAAPGAAAAGVEAGSKGNCAVWTFRGSWQLPGDVEADGVAAALERGVLRVTLPRRQAPEKPQPRRIKVTAGAQ

>CrHSP22D / Cre01.g020575 / gi|158283943|gb|EDP09693|

MALSVTVLHVVNNVAARAGRCQTLPNACVPPQRPQPGHSSPQPALASPSHLARRMLDPARLFLGSSFSPLMDFGFGDLDRYELQAYCPGMAKQDVVVELTPDNVICINGSHKAKLVTPAPQLPKPEAAAAAGEAAANSDAEDPARPAAPAAEAAAAEPEMLGTYRFSRSFGLPEDADV

>CrHSP22E Cre14.g617450 / gi|158283809|gb|EDP09559|

MATTLMKSVASTSGAASARRKTAAPAGAHAIAMRSMLPVRPSRQGAVTPQAFYLSPYACKPARVYRSSPFSLGMGRLASELLRESVTCTHAVDISALEDRYVLTSDCPGMSEEDISVEISPERVLTIAGARKANPLHQQPRPAATPKPEPQAAAADGDDAASDDAPAAAPSNPERAVRVSYRFSRSFGLPEDAEVEGVSASLDRGVLTVTVPRRVVDKPKPRRVSVSAAAGTAAPEPPSTA

>CrHSP22F / Cre14.g617400

MATTLMKSVASTSGAASARRKTAAPAGARAIAMRSMLPVRPSRQGAMTPQAFYLNPYACKPARVYRSSPFSLGMGRLASELLRESAACTHAVDISALEDRYVLTSDCPGMSEEDISVEISPERVLTIAGARKANPLHQQPRPAATSKPEPLAAAVDGDDAASDDAPAAAPSPPEPAVRVSYRFSRSFGLPEDAEVEGVSASLDRGVLTVTVPRRAVDKPKPRRVSVSAAAGTAAPEPPSTA

>CrHSP22G / Cre13.g572350

MLLRTVATAAAVRGQAPAASRRAGSCAAPATAATSAAATALRARGAALLAAPQLRRGIFTRTGATGKDEQQAATAATGVGSGGGGAGTEAQQQSQQQQQQQQQTAMAPAMGGRGGELDMLSGTLLPSPFRRMADHMMQMQREMDDMLCTFAAPLLGPSAALSPTDPFMLDLFAEPTATALPLQPLSRRGGGAPSLAATAGGGGLAARLLGGGRLVPVVEVEERESEYVVTAEVPGFDKNEIKVSLSDDGVLTMTGTHAESSETAPTQPQPSAASGSEAPAAEQQQTVPTPQGIPGTSKRPSGAPSTGSSSYSRYSSFVRALRLPVGGVEPEGIKAATQHGVLTVTIPKKAQPVPKVREIPVA

>CrHSP22H / Cre07.g318600

MALMLSDPFTNEIDRAMNRMLSSFGVPVQRGGGGGGAIMPGAMDLWKPFTSGMGGGTTTMPMDIIETPEAFELHCDTPGMNPDDVKVELHEGVLTVSGGRKVSREDKDVSGKVWRAERSSFSFSRAFTLPDNAQADSICASMDNGVLKVCIPKKEVEKVEPKRIAITGGGGQTTHMLQQPAHMGGGGQHMQHGAGGRMHQTGKQGGGGGGMMEGGTGGAGGGTTGTGTTGGTA

>VcHSP22A / Vocar.0003s0067 / gi|300260490|gb|EFJ44709|

MALSTVVFADPFFSEMDRAMNRFINSALGNPMSGATAGGSSRAGVAQPSLAMDIIETPTAYELHADTPGMSPEDVKVELHEGVLTVSGERKISHSLKDEGGKVWRSERSSYSFSRAFTLPENANAEDISASIDKGVLRVTVPKKEPPAKKEPKRIAVKSAL

>VcHSP22B / Vocar.0003s0054 / gi|300260416|gb|EFJ44635|

MALSTVVFADPFFSEMDRAMNRFINSALGNPMSSATAGGSSRAGVAQPTLAMDIIETPTAYELHADTPGMTPEDVKVELHEGVLTVSGERKISHSLKDEGGKVWRSERSSYSFSRAFTLPENANAEDISASINKGVLRVTVPKKEPPAKKEPKRIAVKSAL

>VcHSP22C / Vocar.0003s0062 / gi|300260422|gb|EFJ44641|

MALTLFNDPFMTEMDRAMNRMLSSFGMPTTRGTATTSMPMDIFRPFTGTTSGATTMPMDIIETPTAYELHADTPGMTPEDVKVELHEGVLTVSGNRKVAREEKDAQGKVWRSERSSYSFARSFTLPENVNSDNICATIDKGVLKVCVPKKETEPKPEPKRITVTGA

>VcHSP22D / Vocar.0018s0065 / gi|300265242|gb|EFJ49434|

MALTLFNDPFMTEMDRTMNRMLSSFGIPTSRGTATANWPVDILRPFTGTTTGATSMPMDIIETPTAYELHADTPGMAPEDVKVELHEGVLTVSGNRKIAREEKDAQGKVWRSERSSYSFARSFTLPENVNSDNICATIDKGVLKVCVPKKETEPKPEPKRITVTGA

>VcHSP22E / gi|300268357|gb|EFJ52538|

MQFLALRCGCLMRMPRCKCLDVDASMGPEDIRVDVSPDRILTIAGSRQMTRIRSAAGEAAAATVAEAEAGHSSHDDKQAASEKVPPARKSTRGNGGPCSRVASYRFSRSFVLPDDADVGGVAAALERGVLTITIPRRAMGGPKTRRVKISVSDKGDR

>VcHSP22F / Vocar.0001s0181

MFLSSLSGRVAPFVRKSSVAMLFTASHRPLFAASSARRVTAAPVAAPIAPGARSFASILSRAQKEPGQPQDAGAQKKSEQPEHTSGLQPRTPQQSGDLSMMPLGTMMPTPFRRMADQMLQMQREMESLMGAFGMPSSMLATDPWDMFDRAAAPLVTRRAGALGRVVPLEVSEDDKSYMVTAEVPGFDKNKIKISVSEEGVLTMTGSLEEGSLEQGEGEGGMEKGKEGAVKAGTRRYASFVRSIQLPDDANMENITANTQHGVLTVRIPKAAKPAPKVREIPIS

>VcHSP22G / gi|300268838|gb|EFJ53018|

MAPKDPQKLQQQQEQRKQPPQRRSWASAVASLAKRVVDPLGIFSRGFPRSATQRSNRSGDAGAAAATAALPAADTSAAATAEAADTPAAFHAAEWRRDLVAAAAEEDDEAVTAAETAEATAAAALSEDLANDVAMGEVADEEGEEGEGESSHDRGTAAAEHGGVSDEGGSGGCSAGGNGAAAARRHEAATPVLGKHTPLGGGRHVYKSRPPATAYPVDIYEDEDSYELQVVGRYTLCDICAQADVPGMREGDLAVEILDRHRLVLEGSAVVAATERVRPLTVAEVAVAEALLPPGRRPRALRTERRRRRHFKRTFRLPHDVDPSAVTASLQDGVLIVRIAKLRTAGSGSGGDGGAAASSLSFQRRIRVERLPAAVAAADPRDVAAVAAEAAAAARSDALEPPPPPVVGAAAPAPLPTLRDSSATPPPPPPLLLPEFAVPPAVKPPLPPPPPSSLTTPAVPKMQVLVPPQTWASMLPLGTLMSLSYSSMLGVSSGQNGAGGAGGNGDDAATVGSGRDGGMRTEARSPMRPPVPPMTPRQPK

>GpHSP22A / gi|1004139804|gb|KXZ47807|

MALSTVFADPFFTDVDRVMNRMIGNALGSSLLGNSAVARSGAASHSPSLHAMDILETPQSYELHADTPGLAPEDVKVELQEGVLTVSGERKVSHQTSGGKVWRSERSSYSFSRSFTLPENADIDRITASVDKGVLTVIVPKKEPPAKPEPKRITVTGA

>GpHSP22B / gi|1004139805|gb|KXZ47808| MDILETPQAFELYADTPGLAPEDITVELQEGILIVSGERKVSLQTSGGKVWCSERASYSFSRSLALPESADADRITASVDKGVLTVIVPKKEPPAKPEPKRITVTGAA

>GpHSP22C / gi|1004139809|gb|KXZ47812|

MALTLFNDPFMTEMDRTMNRMLNTFMPGMGGWGTTTGTRGTTAGPMDLWRGTTGTTITMPMDIIETPQAFELHADTPGMTPDDIKVELHEGVLTLSGNRKVTRDEKDPTGKVWRSERSSYSFARSFTLPDNVDSDNICASMDKGVLKVCVPKKEVEPKAEPKRITVKGEMGSSQMGTIGGATGTTTTTGGTTGTEGRM

>GpHSP22D / gi|1004146267|gb|KXZ54243|

MALTLFNDPFVMDMDRTMTRMLNSLGAISPAGSRPGASRNHASGSFAMPMDIVETSSAYELTADTPGMSPDDLKVELHEGVITLSGSRKIVRDDKDPAGKVWRSERSSYSFSRSFVLPDNADADAISASMDRGVLKLTVPKKEPQPKPQPKRIAVTGA

>GpHSP22E / gi|1004143697|gb|KXZ51679|

MLASEWLREATSSVHPMDIKAFDDRYEIHSDVPGMAEEDVEVEISPDRVLTIAGSRKTARQGPSAPKPSTNEAAGAADDATLAASSDRASTEPAADVAVSYRFRRSFVLPEDAEVEGVAANLQRGVLTVTVPRRVVEKPQPRRVRVKGAK

>GpHSP22F / gi|1004141316|gb|KXZ49309| MICTAATRTPLAALSGRRTAPLHPSTSPAAPPYPRALSILSRAHKDDKPQQQSSSDVPVAKAEEGQKTGAELSTAQQRGGDIMDIAPALMPPPFRQVAIGRMADHMLQMQREMDSLMGAFGMPSPLSLADPFDIFDRAATAPLLARRALAPAARGLLSRPVALEVDEDAEGYTVTAEVPGFDKNEIKISISEDGILTMTGSHEETTVATEAQQQKLEKGDVAAKTPEQAAAGAGKAEGGLPRGGARRYASFVRSVALPDDVDQERVTATTQHGVLTVRIPKSPKPAPKVREIPVA

>At17.4_cytI / AT3G46230
MSLVPSFFGGRRTNVFDPFSLDVWDPFEGFLTPGLTNAPAKDVAAFTNAKVDWRETPEAHVFKADVPGLKKEEVKVEVEDGNILQISGERSSENEEKSDTWHRVERSSGKFMRRFRLPENAKVEEVKASMENGVLSVTVPKVQESKPEVKSVDISG

>At17.6A_cytI / AT1G59860
MSLIPSFFGNNRRINNNIFDPFSLDVWDPFKELQFPSSSSSAIANARVDWKETAEAHVFKADLPGMKKEEVKVEIEDDSVLKISGERHVEKEEKQDTWHRVERSSGGFSRKFRLPENVKMDQVKASMENGVLTVTVPKVETNKKKAQVKSIDISG

>At17.6B_cytI / AT2G29500
MSMIPSFFNNNRRSNIFDPFSLDVWDPFKELTSSSLSRENSAIVNARVDWRETPEAHVFKADLPGLKKEEVKVEIEEDSVLKISGERHVEKEDKNDTWHRVERSSGQFTRRFRLPENVKMDQVKAAMENGVLTVTVPKAETKKADVKSIQISG

>At17.6C_cytI / AT1G53540
MSLIPSIFGGRRTNVFDPFSLDVFDPFEGFLTPSGLANAPAMDVAAFTNAKVDWRETPEAHVFKADLPGLRKEEVKVEVEDGNILQISGERSNENEEKNDKWHRVERSSGKFTRRFRLPENAKMEEIKASMENGVLSVTVPKVPEKKPEVKSIDISG

>At17.8_cytI / AT1G07400
MSLIPSFFGNNRRSNSIFDPFSLDVWDPFKELQFPSSLSGETSAITNARVDWKETAEAHVFKADLPGMKKEEVKVEIEDDSVLKISGERHVEKEEKQDTWHRVERSSGQFSRKFKLPENVKMDQVKASMENGVLTVTVPKVEEAKKKAQVKSIDISG

>At18.1_cytI / AT5G59720
MSLIPSIFGGRRSNVFDPFSQDLWDPFEGFFTPSSALANASTARDVAAFTNARVDWKETPEAHVFKADLPGLKKEEVKVEVEDKNVLQISGERSKENEEKNDKWHRVERASGKFMRRFRLPENAKMEEVKATMENGVLTVVVPKAPEKKPQVKSIDISGAN

>At17.6_cytII / AT5G12020
MDLGRFPIISILEDMLEVPEDHNNEKTRNNPSRVYMRDAKAMAATPADVIEHPNAYAFVVDMPGIKGDEIKVQVENDNVLVVSGERQRENKENEGVKYVRMERRMGKFMRKFQLPENADLDKISAVCHDGVLKVTVQKLPPPEPKKPKTIQVQVA

>At17.7_cytII / AT5G12030
MDLEFGRFPIFSILEDMLEAPEEQTEKTRNNPSRAYMRDAKAMAATPADVIEHPDAYVFAVDMPGIKGDEIQVQIENENVLVVSGKRQRDNKENEGVKFVRMERRMGKFMRKFQLPDNADLEKISAACNDGVLKVTIPKLPPPEPKKPKTIQVQVA

>At17.4_cytIII / AT1G54050
MSAVAINHFFGLPEAIEKLILPISRSGESNNESRGRGSSNNIPIDILESPKEYIFYLDIPGISKSDIQVTVEEERTLVIKSNGKRKRDDDESEEGSKYIRLERRLAQNLVKKFRLPEDADMASVTAKYQEGVLTVVIKKLPPQPPKPKTVQIAVS

>At15.4_cytIV / AT4G21870
MDFQTIQVMPWEYVLASQSLNNYQENHVRWSQSPDSHTFSVDLPGLRKEEIKVEIEDSIYLIIRTEATPMSPPDQPLKTFKRKFRLPESIDMIGISAGYEDGVLTVIVPKRIMTRRLIDPSDVPESLQLLARAA

>At21.7_cytV / AT5G54660
MTSSSGSLKLEIHTDDKTPGKWSVPLGDDVFRRFLSGGGGSEKAVFGEGSLFSPFLFGKYFDPSDAFPLWEFEAEVLLASLRSLGQCRVDWSQTDQAYVLKSDIPVVGKNNVQVYVDINGRVMEISGQWNSNKKAATNSDWRSGRWWEHGYVRRLELPSDADAKYSEAFLSNNDDYSFLEIRIPKINSKNKF

>At18.5_cytVI / AT2G19310
MSMIPISNRRRLSPGDRIWEPFELMNTFLDFPSPALFLSHHFPSLSREIFPQTSSSTVNTQLNWTETPTAHVFKAYLPGVDQDEVIAFVDEEGYLQICTGDNKFMSRFKLPNNALTDQVTAWMEDEFLVVFVEKDASSSPPQLPEIEENRNVRVVEITGDDD

>At15.7_px / AT5G37670
MADRGIFLYPFRRFQEWSRSTALIDWMESNNSHIFKINVPGYNKEDIKVQIEEGNVLSIRGEGIKEEKKENLVWHVAEREAFSGGGSEFLRRIELPENVKVDQVKAYVENGVLTVVVPKDTSSKSSKVRNVNITSKL

>At22.0_er / AT4G10250
MMKHLLSIFFIGALLLGNIKTSEGSLSSALETTPGSLLSDLWLDRFPDPFKILERIPLGLERDTSVALSPARVDWKETAEGHEIMLDIPGLKKDEVKIEVEENGVLRVSGERKREEEKKGDQWHRVERSYGKFWRQFKLPDNVDMESVKAKLENGVLTINLTKLSPEKVKGPRVVNIAAEEDQTAKISSSESKEL

>At21_cp / AT4G27670
MASTLSFAASALCSPLAPSPSVSSKSATPFSVSFPRKIPSRIRAQDQRENSIDVVQQGQQKGNQGSSVEKRPQQRLTMDVSPFGLLDPLSPMRTMRQMLDTMDRMFEDTMPVSGRNRGGSGVSEIRAPWDIKEEEHEIKMRFDMPGLSKEDVKISVEDNVLVIKGEQKKEDSDDSWSGRSVSSYGTRLQLPDNCEKDKIKAELKNGVLFITIPKTKVERKVIDVQIQ

>At23.5_mt / AT5G51440
MASSSALALRRLLSSSTVAVPRALRAVRPVAASSRLFNTNAARNYEDGVDRNHHSNRHVSRHGGDFFSHILDPFTPTRSLSQMLNFMDQVSEIPLVSATRGMGASGVRRGWNVKEKDDALHLRIDMPGLSREDVKLALEQNTLVIRGEGETEEGEDVSGDGRRFTSRIELPEKVYKTDEIKAEMKNGVLKVVIPKIKEDERNNIRHINVD

>At23.6_mt / AT4G25200
MASALALKRLLSSSIAPRSRSVLRPAVSSRLFNTNAVRSYDDDGENGDGVDLYRRSVPRRRGDFFSDVFDPFSPTRSVSQVLNLMDQFMENPLLSATRGMGASGARRGWDIKEKDDALYLRIDMPGLSREDVKLALEQDTLVIRGEGKNEEDGGEEGESGNRRFTSRIGLPDKIYKIDEIKAEMKNGVLKVVIPKMKEQERNDVRQIEIN

>At26.5_mtII / AT1G52560
MALARLALRNLQQKLSPSLMGQSCERGLVGNRHNPMKLNRFMATSAGEQEDKMNTEVSVSEKKSPRQNFPRRRGRKSLWRNTDDHGYFTPTLNEFFPPTIGNTLIQATENMNRIFDNFNVNPFQLMGQVKEQDDCYKLRYEVPGLTKEDVKITVNDGILTIKGDHKAEEEKGSPEEDEYWSSKSYGYYNTSLSLPDDAKVEDIKAELKNGVLNLVIPRTEKPKKNVQEISVE

>At14.2 / AT5G47600
MSRNMEVNAGSSGEIPSPIRNRFQKSGSQAVYEVTETKKSCVTRVDMPGCPESDLTYWVDANNVHFFADEPAMPEYENAGRKYGGSMIFNPEAYDVKKTKVKLINGVLWITVPKIPGKNASINVKERILHY
